# Wnt Signaling in Atherosclerosis: Mechanisms to Therapeutic Implications

**DOI:** 10.3390/biomedicines12020276

**Published:** 2024-01-25

**Authors:** Rizwana Afroz, Julie E. Goodwin

**Affiliations:** 1 Department of Pediatrics, Yale University School of Medicine, New Haven, CT 06520, USA; rizwana.afroz@yale.edu; 2 Vascular Biology and Therapeutics Program, Yale University School of Medicine, New Haven, CT 06520, USA; 3 Department of Cellular and Molecular Physiology, Yale University School of Medicine, New Haven, CT 06520, USA

**Keywords:** Wnt pathway, atherosclerosis, endothelial dysfunction, Wnt inhibitors

## Abstract

Atherosclerosis is a vascular disease in which inflammation plays a pivotal role. Receptor-mediated signaling pathways regulate vascular inflammation and the pathophysiology of atherosclerosis. Emerging evidence has revealed the role of the Wnt pathway in atherosclerosis progression. The Wnt pathway influences almost all stages of atherosclerosis progression, including endothelial dysfunction, monocyte infiltration, smooth muscle cell proliferation and migration, and plaque formation. Targeting the Wnt pathway to treat atherosclerosis represents a promising therapeutic approach that remains understudied. Blocking Wnt signaling utilizing small molecule inhibitors, recombinant proteins, and/or neutralizing antibodies ameliorates atherosclerosis in preclinical models. The Wnt pathway can be potentially manipulated through targeting Wnt ligands, receptors, co-receptors, and downstream signaling molecules. However, there are challenges associated with developing a real world therapeutic compound that targets the Wnt pathway. This review focuses on the role of Wnt signaling in atherosclerosis development, and the rationale for targeting this pathway for the treatment of atherosclerosis.

## 1. Introduction

Atherosclerosis is a disease of the blood vessels and is a major underlying cause for a broad spectrum of cardiovascular conditions such as ischemia, myocardial infarction (MI), and stroke [1]. This disease is characterized by the accumulation of lipids, inflammatory cells, and extracellular matrix (ECM) components in the intimal region of the large arteries, contributing to the progressive thickening of the intima. Several risk factors and mechanisms are associated with the development of atherosclerosis. Until the 1970s, the pathophysiology of atherosclerosis was predominantly focused on its link with lipids. Subsequently, emerging knowledge of vascular biology gradually identified inflammation as the key player in the process of atherogenesis [1,2]. Several signaling pathways involved in inflammation have been implicated in atherosclerosis. The involvement of Wnt signaling in cardiovascular disease was first reported in an Iranian family with autosomal dominant early coronary artery disease (CAD), features of metabolic syndrome (hypertension, hyperlipidemia, and diabetes), and osteoporosis. A missense mutation in the Wnt co-receptor low-density-lipoprotein related protein 6 (LRP6) was shown to be linked with these traits [3]. Since then, a common functional variant of LRP6 has been identified as an independent risk factor for carotid artery atherosclerosis in hypertensive patients [4]. Several studies indicated the presence of a Wnt ligand, Wnt5a, in the intimal areas of macrophage accumulation [5], and reported a correlation between Wnt5a expression and severity of atherosclerotic lesion [6].

Wnt signaling influences atherosclerosis development at all stages, from endothelial dysfunction to plaque formation [7]. In this review, we aim to explore the complex role of Wnt signaling in atherosclerosis. We provide a brief summary of the Wnt pathway, followed by a comprehensive description of its role in atherosclerosis development. Finally, we discuss the therapeutic opportunities and challenges of targeting Wnt signaling for the treatment of atherosclerosis.

## 2. The Wnt Signaling Axis

**Figure 1:**
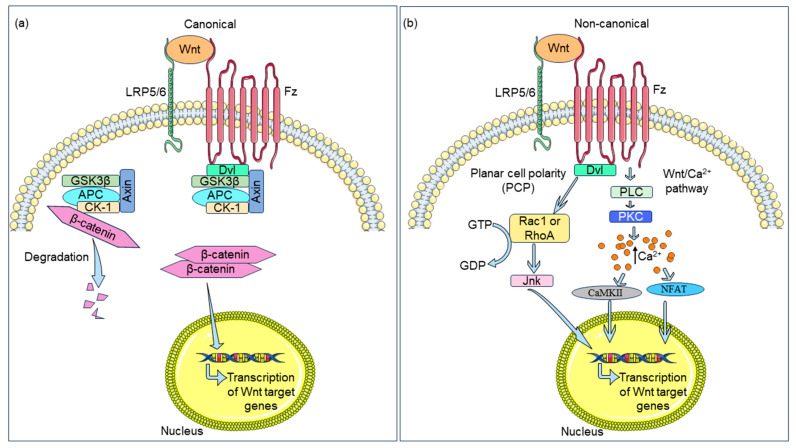
Schematic overview of the Wnt pathway. (**a**) In the absence of Wnt stimulus, β-catenin remains attached to a destruction complex and degrades. Wnt binding to the frizzled (Fz) receptor recruits disheveled (Dvl) protein which binds to axin and other proteins of the β-catenin destruction complex. This results in the release of β-catenin from the destruction complex. β-catenin translocates to the nucleus and enables transcription of Wnt target genes. (**b**) In non-canonical Wnt signaling, Wnt ligand binding activates Dvl protein. Dvl protein facilitates GTP-dependent activation of the small GTPases RhoA and Rac1, which leads to the phosphorylation and activation of JUN N-terminal kinase (Jnk) and eventual transcription of Wnt target genes. The non-canonical Wnt pathway also elevates the intracellular Ca^2+^ concentration via activation of phospholipase C (PLC) and protein kinase C (PKC). Elevated Ca^2+^ concentration results in the activation of calcium/calmodulin-dependent protein kinase II (CaMKII) and nuclear factor of activated T cells (NFAT), leading to the transcription of Wnt target genes [15,20]. Components of this figure were created using Servier Medical Art templates, which are licensed under a Creative Commons Attribution 3.0 Unported License; https://smart.servier.com (accessed on 15 November 2023).

Wnt signaling is an evolutionarily conserved pathway which regulates proliferation, survival, differentiation, cell fate specification, and organogenesis [8,9,10]. Aberrant Wnt signaling is linked to a variety of pathologies, including atherosclerosis [11,12]. In mammals, the Wnt pathway is composed of nineteen signaling molecules and ten frizzled (Fz) receptors. The Fz receptors contain a seven transmembrane spanning domain, an extracellular cysteine-rich N-terminal domain, and a cytoplasmic tail. LRP5 and LRP6 serve as co-receptors to these Fz receptors in the signaling complex [13,14]. Wnt signaling can be classified into two key axes: the canonical (β-catenin dependent) and the non-canonical (β-catenin independent) arms [15,16]. Β-catenin, the key component of canonical Wnt signaling, remains phosphorylated via a multiprotein destruction complex in the absence of Wnt stimulus. Phosphorylated β-catenin undergoes poly-ubiquitination and subsequent proteasome-mediated degradation. Binding of a Wnt ligand to the Fz receptor complex facilitates the recruitment of disheveled (Dvl) protein and axin, resulting in the release of β-catenin from the destruction complex. Cytoplasmic accumulation of β-catenin allows for its nuclear translocation, and this enables β-catenin to regulate the transcription of an array of genes involved in development and disease [17] (Figure 1a).

Wnt ligands also exert cellular effects without involving β-catenin; this is known as non-canonical Wnt signaling. Planar cell polarity (PCP) and Wnt/Ca^2+^ signaling are the two major classes of non-canonical Wnt signaling. Dvl proteins are activated by the binding of a Wnt ligand to either Fz, a receptor tyrosine kinase-like orphan receptor (ROR), or a related receptor tyrosine kinase (RYK). Dvl facilitates GTP-dependent activation of the small GTPases RhoA and Rac1, which leads to the phosphorylation and activation of JUN N-terminal kinase (Jnk) and the eventual transcription of Wnt target genes [15,18]. Wnt ligands also regulate intracellular Ca^2+^ concentration. The Wnt/Ca^2+^ pathway involves activation of phospholipase C (PLC) and protein kinase C (PKC), which elevates the intracellular Ca^2+^ concentration. Increased Ca^2+^ concentration is responsible for activating calcium/calmodulin-dependent protein kinase II (CaMKII) and the nuclear factor of activated T cells (NFAT), which leads to the transcriptional regulation of Wnt target genes [15,16,19] (Figure 1b).

## 3. Wnt Signaling Influences Endothelial Dysfunction

The inner linings of the blood vessels are made up of single layers of endothelial cells (the endothelium) that are in direct contact with the blood and circulating cells. Endothelial dysfunction is the earliest hallmark of the pathogenesis of atherosclerosis [1]. Normal endothelium resists prolonged contact with blood leukocytes, including monocytes. Under pathological circumstances, the arterial endothelium undergoes inflammatory changes, allowing blood monocytes to adhere. These monocytes differentiate into macrophages that internalize cholesterol-containing low-density lipoproteins (LDLs) and form foam cells. Moreover, these macrophages release various cytokines that trigger smooth muscle cell migration from media to the intima. Both lipid-laden macrophages and smooth muscle cells contribute to the formation of atherosclerotic plaques with a fibrous cap on the luminal side of a vessel. Rupture of this fibrous cap can release the plaque components into the arterial lumen, resulting in thrombus formation in the artery. Thrombus formation is the ultimate complication of an atherosclerotic lesion and can occlude a vessel [2].

**Figure 2:**
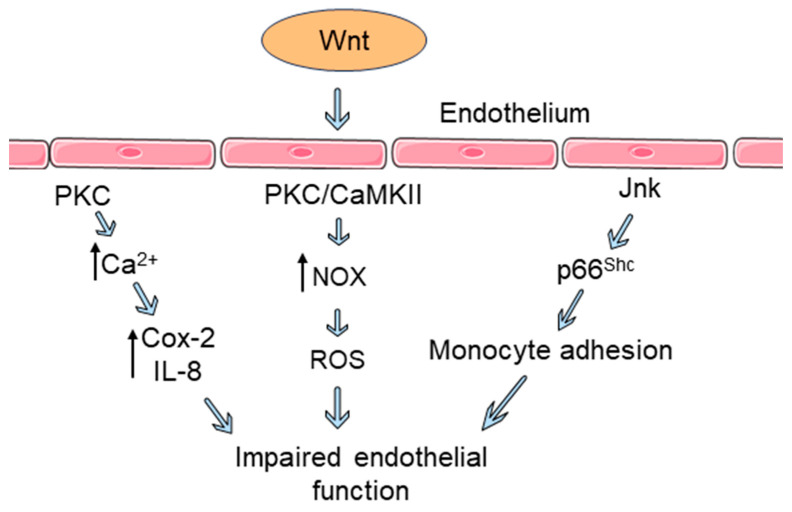
Wnt signaling in endothelial dysfunction. The Wnt pathway influences endothelial dysfunction through diverse mechanisms. Wnt ligand binding to endothelial cells triggers cyclooxygenase-2 (Cox-2) expression and the release of inflammatory cytokines via Ca^2+^/protein kinase C (PKC) signaling, which contributes to increased endothelial permeability. In endothelial cells, Wnt signaling triggers NADPH oxidase (NOX) activation, leading to the increased production of reactive oxygen species (ROS). Both PKC and calcium/calmodulin-dependent protein kinase II (CaMKII) interact with NOX and possibly regulate vascular oxidative stress. Binding of a Wnt ligand induces the phosphorylation and activation of redox regulatory protein p66^Shc^ by Jnk in endothelial cells, which triggers monocyte adhesion to the endothelium. These processes contribute to increased endothelial permeability and impaired endothelial barrier function. Components were created using Servier Medical Art templates, which are licensed under a Creative Commons Attribution 3.0 Unported License; https://smart.servier.com (accessed on 27 December 2023).

Endothelial cells express a large panel of Fz receptors, and Wnt signaling regulates the roles and functions of these cells [21]. Wnt signaling influences endothelial dysfunction via diverse mechanisms (Figure 2). Increased expression of Wnt5a was reported in primary venous endothelial cells isolated from patients with diabetes; higher levels of Jnk phosphorylation and activation were also observed. Higher Jnk activation in endothelial cells was associated with lower flow-mediated dilation, an indicator of endothelial dysfunction. Inhibition of Wnt5a-induced Jnk activation restored impaired endothelial nitric oxide synthase (eNOS) activation and nitric oxide production (NO) in human endothelial cells [22]. Wnt5a induces rapid expression of cyclooxygenase-2 (Cox-2) and upregulation of inflammatory cytokines, particularly interleukin-8 (IL-8), in human aortic endothelial cells (HAECs). Moreover, Wnt5a treatment increases HAEC monolayer permeability, a hallmark of early endothelial inflammation and barrier dysfunction. PKC and Ca^2+^ were involved in this response, which reveals the role of non-canonical Wnt signaling in endothelial inflammatory regulation [23]. Crosstalk between Wnt/β-catenin signaling and TNF-α/NF-κB signaling regulates various aspects of endothelial dysfunction. Inhibition of β-catenin activity via an inhibitor of β-catenin-responsive transcription-14 (iCRT-14) suppresses TNF-α-mediated expression of NF-κB, along with decreased production of cytokines in cultured endothelial cells. In the presence of iCRT-14, TNF-α-induced monocyte adhesion and upregulation of vascular cell adhesion molecule-1 (VCAM-1) was reversed in human umbilical vein endothelial cells (HUVECs) and human coronary artery endothelial cells (HCAECs). Interruption in Wnt/β-catenin signaling with iCRT-14 also lowered endothelial permeability to restore endothelial barrier function [17].

Wnt3a induces the phosphorylation and activation of redox regulatory protein p66^Shc^ in endothelial cells. Knockdown of p66^Shc^ suppresses Wnt3a-mediated β-catenin release and attenuates β-catenin dependent transcription. Wnt3a impairs endothelium-dependent vasorelaxation in mouse aorta, which is reversed through p66^Shc^ knockdown. Moreover, p66^Shc^ regulates monocyte adhesion to Wnt3a-stimulated endothelial cells. Canonical Wnt signaling influences endothelial oxidative stress and dysfunction, in which p66^Shc^ plays a pivotal role [9]. Wnt5a acts as an effector of IL-4 induced hyperpermeability of vascular endothelial cells (which leads to endothelial barrier dysfunction) [24]. Assembly of adherent junction proteins (β-catenin and VE-cadherin) is disrupted when HCAEC monolayers are treated with Wnt5a. Small holes and gaps are found between cells in the monolayer as β-catenin and VE-cadherin proteins are lost at cell borders. In an Electric Cell-substrate Impedance Sensing (ECIS) experiment, the real-time trans-endothelial electric resistance (TEER) of the HCAEC monolayer was decreased, which indicates increased endothelial permeability and impaired endothelial barrier function. Silencing of RYK reversed the Wnt5a-induced endothelial hyperpermeability, and thus revealed the role of Wnt5a/RYK signaling in endothelial dysfunction [25].

A particular mutant LRP6 (p.Y418H) was reported to contribute to normolipidemic familial CAD via impairing normal functioning of endothelial cells [26]. The LRP6 Y418H mutation weakened Wnt signal transduction with altered proliferation, migration, and survival properties in HUVECs. Endothelial integrity depends on homeostasis and prompt renewal of endothelial cells. The impaired endothelium associated with the LRP6 Y418H mutation increases the risks of developing atherosclerosis in coronary arteries [26]. Wnt signaling regulates endothelial cell proliferation either via the canonical (β-catenin dependent) or the non-canonical (β-catenin independent) pathway [21,27]. In endothelial cells, non-canonical Wnt signaling triggers NADPH oxidase (NOX) activation which contributes to increased reactive oxygen species (ROS) production [28,29,30]. Both PKC and CaMKII interact with NOX and possibly regulate vascular oxidative stress [15]. This observation indicates the possible regulation of vascular oxidative stress by non-canonical Wnt signaling in endothelial cells [15].

Wnt signaling plays a pivotal role in interrupting the normal functioning of endothelium. Emerging evidence suggests the linked involvement of both canonical and non-canonical Wnt signaling in endothelial dysfunction. Activation of aberrant Wnt signaling can trigger multiple interrelated pathways that induce functional changes to the endothelium and initiate the process of endothelial dysfunction.

## 4. Wnt Pathway Regulates Vascular Smooth Muscle Cell Proliferation and Migration

Vascular smooth muscle cells (VSMC) are located in the media in healthy arteries and are responsible for arterial contraction and production of ECM. In early atherosclerosis, VSMCs migrate towards the intima and their contractile phenotype changes to a proliferative synthetic phenotype. Synthetic VSMCs produce ECM rich in proteoglycans and glycosaminoglycans, which retain more lipids in the vessel wall [31,32].

VSMC activation is a complex process and a critical step in atherosclerosis development. Activation of VSMCs results in the proliferation and migration of these cells. The Wnt/β-catenin pathway triggers the proliferation of mouse aortic VSMCs. Pharmacological inhibition of the Wnt/β-catenin pathway by Wnt inhibitory factor-1 (WIF-1) reduces VSMC proliferation in vitro. siRNA knockdown of Wnt2 and Wnt4 also results in decreased proliferation of VSMCs. Wnt4 upregulation activated the Wnt/β-catenin pathway and stimulated the proliferation of VSMCs during intimal thickening in a transgenic mouse model in which left carotid artery ligation was performed to induce intimal thickening [33]. Hyperlipidemia also induced VSMC proliferation within the thoracic aorta of high fat diet-fed rats. Increased expression of Wnt3a, β-catenin, cyclin D1, and other Wnt target genes were associated with the proliferation of rat aortic VSMCs [34].

**Figure 3:**
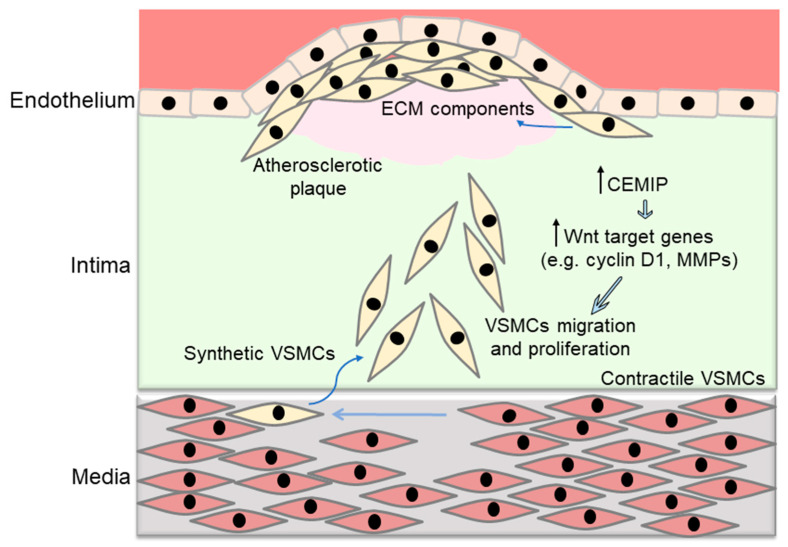
Wnt signaling regulates vascular smooth muscle cell (VSMC) proliferation and migration. VSMCs reside in the media within healthy arteries. In early atherosclerosis, the contractile phenotype of VSMCs changes to a synthetic phenotype and VSMCs migrate towards the intima. Synthetic VSMCs produce extracellular matrix (ECM) rich in proteoglycans and glycosaminoglycans, which retain more lipids in the vessel wall. Pharmacological and genetic inhibition of the Wnt pathway reduces VSMC proliferation. Overexpression of cell-migration-inducing and hyaluronan-binding protein (CEMIP) promotes VSMC proliferation and migration via activating the Wnt/β-catenin pathway and the upregulation of Wnt target genes including matrix metalloproteinases (MMPs), cyclin D1, and others.

Cell-migration-inducing and hyaluronan-binding protein (CEMIP) is known to bind and degrade hyaluronic acid and is involved in remodeling of the ECM [35]. Overexpression of CEMIP at both the mRNA and protein levels have been observed in plasma samples from atherosclerosis patients. Overexpressed CEMIP induces the migration and proliferation of VSMCs. CEMIP promotes VSMC proliferation and migration via activating the Wnt/β-catenin pathway and through the upregulation of Wnt target genes, including matrix metalloproteinases (MMPs), cycling D1, and others [36] (Figure 3). Human recombinant Wnt5a was shown to induce VSMC migration in a wound healing/scratch assay, in which human VSMCs migrated significantly after 18 and 24 h as compared to non-treated control cells. Although Wnt5a has been shown to inhibit VSMC proliferation, Wnt3a has been found to stimulate VSMC proliferation compared to control cells. Human VSMCs treated with Wnt5a showed increased lipid droplet deposition within the cytoplasm after LipidTox. Pre-treatment of VSMCs with the Wnt5a competitive inhibitor Box5 significantly suppressed the Wnt5a-induced lipid droplet accumulation [37]. Wnt3a treatment was shown to increase the proliferation of VSMCs from young adult rats, along with increasing the expression of cycling D1 mRNA and protein levels [38]. A microRNA (miRNA) expression profile study in patients with stable and unstable carotid plaques revealed that miR-210 is most significantly downregulated in local plasma and tissue from the fibrous cap in these patients. miR-210 stimulates Wnt signaling via blocking adenomatous polyposis coli (APC), which leads to the proliferation and survival of VSMCs. APC is part of a destruction complex that prevents nuclear translocation of β-catenin. Inhibition of APC facilitates the activation of the Wnt/β-catenin pathway in VSMC and thus promotes the proliferation and survival of VSMCs in the fibrous cap of atherosclerotic plaques [39].

In the process of atherosclerosis development, endothelial dysfunction and associated inflammation result in the activation of VSMCs. Activated VSMCs subsequently migrate into the intima, where they proliferate and produce lipid-retentive ECM. In addition to endothelial dysfunction and associated inflammatory cascades, Wnt signaling also regulates the proliferation and migration of VSMCs and therefore further contributes to the development of atherosclerosis.

## 5. Wnt Signaling in Macrophages

The importance of inflammation in atherosclerosis was underappreciated for many years. Since atherosclerosis is recognized as an inflammatory disease, macrophages are identified as key players. Endothelial dysfunction allows adhesion, rolling. and migration of monocytes to the subendothelial layers. These blood borne monocytes differentiate into tissue macrophages which scavenge cholesterol-containing LDLs to become foam cells. The formation of foam cells is a characteristic feature for atheromatous lesions. Foam cells release proinflammatory cytokines, ROS, and MMPs, and lead to an amplification of the local inflammatory response and weakening of atherosclerotic plaques [2,21].

Elevated levels of Wnt5a were found in macrophage-rich areas of atherosclerotic plaques [5]. Oxidized LDL (oxLDL) has been reported to induce mRNA expression of Wnt5a in human macrophages [40] and oxLDL and bacterial structures can stimulate Wnt5a release in macrophages. Fz5 and Wnt5a cooperate to initiate the release of proinflammatory cytokines to further enhance local inflammation [41]. Both Wnt3a and Wnt5a activate the NF-κB pathway in murine and human macrophages. However, Wnt3a-mediated activation of the NF-κB pathway is transient, while Wnt5a shows a relatively prolonged and sustained effect on the NF-κB pathway [42]. The expression of LRP5 increases in macrophages in advanced plaques compared to early lesions. LRP5 mRNA and protein levels increase when human monocytes and macrophages are exposed to aggregated LDLs, inducing the Wnt/β-catenin pathway. siRNA silencing of LRP5 suppresses lipid retention in human macrophages after exposure to aggregated LDLs [43]. Macrophages play a pivotal role in inflammation, lipid uptake, and plaque destabilization in atherosclerosis. Therefore, Wnt mediated regulation of macrophages has high importance in the pathophysiology of atherosclerosis. Targeting Wnt signaling in plaque macrophages might be a relevant approach for the treatment of atherosclerosis.

## 6. The Wnt Pathway in Cholesterol Storage and Trafficking

Mammalian cells can synthesize and produce cholesterol, but they primarily acquire cholesterol via endocytosis of LDLs by LDL receptors (LDLR). LDL particles are internalized and transported to the late endosomes, where esterified cholesterol is released and de-esterified. Following this, the free cholesterol is transported to the endoplasmic reticulum and then to the rest of the cells.

The Wnt pathway takes part in cholesterol storage and trafficking in mammalian cells [44]. Mutations in the Wnt co-receptor LRP6 are associated with high cholesterol levels and elevated concentrations of triglycerides and glucose during fasting, thus providing a basis for the development of atherosclerosis [3]. Modifications to LRP6 were found to induce increased cholesterol synthesis and steatosis in the liver [45]. LDLR internalization and LDL uptake were found to be compromised in fibroblasts of LRP6-R611C mutation carriers. This mutation is linked to increased LDL synthesis, de novo lipogenesis, and VLDL secretion in mice. Wnt co-receptor LRP6 has been identified as a receptor for LDL endocytosis and clearance; it was found that internalization of LDLR was markedly suppressed after LRP6 knock down and was restored after LRP6 was reintroduced [46]. LRP6-R611C mutation causes elevated plasma LDL and triglyceride levels along with fatty liver in mice. This mutation also triggers hepatic de novo lipogenesis, lipid and cholesterol biosynthesis, and apoB secretion [45]. LDL uptake is decreased in the splenic B cells of LRP6^+/−^ mice compared to their wildtype littermates [20,47].

## 7. Targeting Wnt Pathways in Atherosclerosis: Therapeutic Opportunities and Challenges

Wnt signaling involves a complex network of pathways that impact vascular inflammation, oxidative stress, endothelial dysfunction and homeostasis, all of which contribute to the progression of atherosclerosis [15]. Both canonical and non-canonical Wnt signaling are mechanistically linked with the pathophysiology of atherosclerosis. In this section of the review, we discuss the opportunities and challenges surrounding targeting the Wnt pathway to treat atherosclerosis.

### 7.1. Potential Strategies to Target Wnt Pathway

Early attempts to target Wnt signaling as a therapeutic approach primarily focused on the treatment of different types of cancer [48,49,50]. However, similar targeting strategies can be utilized for the treatment of atherosclerosis and other cardiovascular diseases. Identifying small-molecule modulators of Wnt signaling looks to be a promising strategy to treat multiple diseases; however, only a few selective candidates have shown remarkable effects. Porcupine inhibitor GNF-6231 is one such example that can modulate the Wnt pathway. GNF-6231 is an endoplasmic reticulum protein which facilitates Wnt palmitoylation. GNF-6231 blocks the secretion of Wnt ligands and therefore inhibits the activation of the Wnt pathway. Pharmacological inhibition of Wnt signaling by post-MI intravenous injection of GNF-6231 in mice preserved cardiac function, prevented detrimental cardiac remodeling, and decreased infarct size. GNF-6231 treatment blocks the activation of the Wnt/β-catenin pathway in the infarcted heart and improves post-MI recovery in C57BL/6J mice. Temporary and systemic inhibition of the Wnt pathway in infarcted hearts promotes the proliferation of cardiac progenitors and other interstitial cells, but selectively reduces myofibroblast proliferation in the distal myocardium [51] (Table 1).

A small molecule Inhibitor that attenuates the Wnt/β-catenin pathway, XAV939, was identified in a chemical genetic screen [52]. XAV939 promotes β-catenin degradation by stabilizing axin, the rate-limiting component of the β-catenin destruction complex. Quantitative chemical proteomic analysis has revealed that XAV939 inhibits poly ADP-ribose polymerase tankyrase 1 and tankyrase 2. Both tankyrase isoforms interact with a highly conserved domain of axin and promote its degradation. Blocking of tankyrase isoforms by XAV939 results in the stabilization of axin [52]. Small molecule inhibitor XAV939 also inhibits intima formation after carotid artery ligation in C57BL/6 mice and suppresses the proliferation, migration, and apoptosis of rat VSMCs. This anti-atherogenic role of XAV939 is associated with the inhibition of nuclear translocation of β-catenin [53]. Inhibition of tankyrase activity by XAV939 was shown to suppress isoproterenol-induced hyperactivation of the Wnt/β-catenin pathway and provide protection against isoproterenol-induced ventricular dilatation in an adult zebrafish model [54] (Table 1).

LGK974 is a small molecule Wnt inhibitor which is currently in a phase one clinical trial for the treatment of selected solid malignancies (NCT01351103). In a preclinical study, inhibition of the Wnt pathway by LGK974 substantially improved renal fibrosis and restored endothelial glucocorticoid receptor (GR) levels. Treatment with LGK974 decreased extracellular matrix deposition, relative area of fibrosis, collagen accumulation, and glomerulosclerosis in diabetic mice. The levels of inflammatory markers including IL-1β, IL-6, IL-10, TNF-α, and MCP-1 were also suppressed by LGK974 [55]. Endothelial GR knockout mice bred onto an ApoE null background showed worsened atherosclerosis compared to ApoE knockout mice alone. The loss of endothelial GR receptor results in upregulation of the Wnt pathway both in vitro and in vivo [56]. Therefore, blocking the Wnt pathway using LGK974 might also reduce atherosclerosis; however, this possibility warrants further investigations (Table 1).

Pyrvinium, a lipophilic cation of the cyanine dye family, has been used as an safe and effective anti-helminthic for over 70 years to treat pinworms [57]. This FDA-approved small molecule Wnt inhibitor blocks the Wnt pathway via activating the β-catenin destruction complex protein CK1α [58]. The efficacy of pyrvinium was tested against different types of malignancies including intestinal adenomas [59], colorectal cancer [60], breast cancer [61], lung cancer [62], glioblastoma [63], and many others [58]. Pyrvinium showed strong anti-cancer activity against various human cancers both in vitro and in vivo. This compound is in a phase one clinical trial (NTC05055323) for the treatment of pancreatic ductal adenocarcinoma that cannot be removed by surgery. Pyrvinium is reported to promote wound repair and post-MI cardiac remodeling in mice [64]. Administration of pyrvinium was shown to improve cardiac dysfunction by attenuating calcium dyshomeostasis and mitochondrial dysfunction in a septic rat model [65]. These studies suggest the validity of utilizing small molecule Wnt inhibitors for the treatment of atherosclerosis. However, identifying a tissue-specific small molecule Wnt inhibitor with therapeutic efficacy will require optimization.

Attenuation of the Wnt pathway can be achieved through the utilization of biologics such as recombinant proteins and neutralizing antibodies. Secreted frizzled-related protein 4 (SFRP4) is a secreted glycoprotein that has been linked to a variety of cardiovascular diseases [66,67]. Elevated circulating SFRP4 levels have been reported in patients with CAD [66]. SFRP4 transcription was upregulated in rat ischemic heart models. Administration of SFRP4 recombinant protein reduced fibrosis scar size and ameliorated cardiac function after ischemic injury in Sprague-Dawley rats. SFRP4 administration attenuates β-catenin activity and potentially exerts its cardio-protective effect through interfering with the Wnt/β-catenin pathway [67]. Administration of recombinant SFRP4 reduced lipid accumulation in the aortic root of high fat diet fed-Apoe knockout mice. This anti-atherosclerotic effect of SFRP4 is associated with reduced mRNA expression of proinflammatory cytokines such as IL-1β, IL-6, IL-17, and TNF-α. Moreover, the infiltration of macrophages and CD4^+^ T lymphocytes in the aortic root was decreased after SFRP4 administration. SFRP4 reduced oxidative stress by increasing nuclear factor E2-related factor 2 and heme oxygenase-1 expression, glutathione levels, and superoxide dismutase activity in the aortic tissues of Apoe knockout mice. Nox activity and malondialdehyde levels were also downregulated in SFRP4-treated mice. The mRNA expression of Wnt pathway components such as GSK-3β and β-catenin was reduced in aortic tissue from SFRP4-administered mice. On the other hand, Wnt1 overexpression abolished the anti-atherosclerotic effect of SFRP4 in Apoe knockout mice [68]. Overexpression of SFRP4 induced by injection of adenoviral SFRP4 vector in ApoE knockout mice was associated with lowered lipid deposition in the whole aorta and aortic roots of these mice [69] (Table 1).

**Table 1:** List of potential compounds that target Wnt pathway.

Compound	Target(s)	Effect(s)
GNF-6231	Wnt ligands	Temporary and systemic inhibition of the Wnt/β-catenin pathway in the infarcted heart and improvement of post-MI recovery [51]
XAV939	Tankyrase	Inhibits nuclear translocation of β-catenin and suppresses proliferation, migration, and apoptosis of VSMCs [53]
		Suppresses hyperactivation of the Wnt/β-catenin pathway in infarcted heart [54]
LGK974	Wnt ligands	Decreases ECM deposition and collagen accumulation, restores endothelial GR levels, and suppresses the expression of inflammatory cytokines [55]
Pyrvinium	CK1α	Promotes wound repair, post-MI cardiac remodeling [64], and improves cardiac dysfunction [65]
SFRP4	Interaction between β-catenin and CD24	Decreases aortic lipid deposition, suppresses inflammatory cytokines, and downregulates Nox activity [68]
		Reduces fibrotic scar size and ameliorates cardiac function after ischemic injury [67]
Sclerostin	Interaction between LRP5/6 and Fz	Provides protection against atherosclerosis and inflammation through reduction of aortic lipid deposition and downregulation of inflammatory cytokines

Sclerostin is a secreted cysteine-knot protein expressed by osteocytes which regulates bone mineralization. This protein binds to LRP5/6 co-receptors and interferes with their binding to Fz receptors. Sclerostin interferes with the LRP5/6-Fz complex formation and inhibits Wnt pathway activation [70]. Administration of recombinant sclerostin via intraperitoneal injection in ApoE knockout mice provides protection against atherosclerosis and inflammation. Overexpression of sclerostin reduces lipid accumulation in mouse aortas. Plasma concentrations of inflammatory markers such as monocyte chemoattractant protein-1, TNF-α, IL-6, and IL-10 have also been shown to be downregulated in sclerostin overexpressing mice. Moreover, sclerostin overexpression suppresses macrophage infiltration in the suprarenal aorta [71]. Blocking Fz receptors with homologous peptides has provided insights for developing treatment strategies targeting Wnt-Fz interactions (Table 1). UM206, a 13-amino acid peptide fragment of Wnt3a/Wnt5a, blocks Wnt pathway activation. Blocking Fz activity through UM206 administration showed remarkable post-MI cardioprotective activity in mice and pigs [72,73].

Utilization of antisense oligonucleotides, nanoparticles, and gene editing methods such as CRISPR-Cas9 can also allow efficient targeting of the Wnt pathway. However, to date, these strategies have not been validated nor optimized extensively to modulate Wnt signaling for the treatment of cardiovascular diseases. These strategies could be of great interest for developing selective and tissue-specific therapeutics that are clinically feasible [15].

### 7.2. Wnt Signaling Components as Therapeutic Targets

Wnt signaling can be manipulated through blocking ligands, receptors, co-receptors, and downstream signaling molecules. Blocking all Wnt ligands could potentially inhibit all downstream signaling; however, simultaneous targeting of all Wnt ligands would not be practically feasible. Targeting of Wnt5a has shown some promising results in preclinical models [66,67,68,69]. Wnt5a is a ligand of the non-canonical Wnt pathway and blocking Wnt5a theoretically blocks the entire non-canonical Wnt signaling arm. However, practically speaking, the canonical and non-canonical pathways interact and cross-regulate each other. Therefore, suppressing the non-canonical pathway would likely subsequently impact the canonical Wnt pathway [15].

Fz receptors, particularly Fz2 and Fz5, are upregulated in internal mammary arteries of individuals with obesity, indicating relatively higher sensitivity to Wnt ligands [74]. Downregulation of Fz receptors thus represents a promising target point for modulating the Wnt pathway. Fz receptor expression can be downregulated using antisense oligonucleotides [15]. However, to date, this strategy remains hypothetical and needs extensive research validation. Recombinant proteins and synthetic nucleotides can block the interaction between Wnt receptors and co-receptors such as sclerostin [70]. One preclinical study reported a promising anti-atherosclerotic effect of sclerostin [71]; therefore, Wnt co-receptors can also be considered as potential therapeutic targets.

The Wnt pathway activates a range of downstream signaling molecules including Rac1, Jnk, CaMKII, and PKC. Targeting these downstream signaling molecules could reverse the detrimental impacts of Wnt signaling. However, many signaling pathways may also converge on these molecules, and thus targeting downstream signaling molecules may cause unexpected side effects [15]. Despite these limitations, studies targeting downstream signaling molecules have reported positive results in animal models. Rac1 deficiency inhibits Nox activation and ROS production and reduces myocardial remodeling in mice with type 1 diabetes [75]. CaMKII inhibition reduces infarct size in irreversible ischemia-reperfusion injury in rats [76]. Chronic CaMKII suppression reverses cardiac function in mice with heart failure [77]. Inhibition of Jnk2 activity reduces atherosclerosis in high fat diet-fed Jnk2 knockout mice [78]. Adipose tissue-specific genetic inactivation of Jnk reduces inflammation in adipose tissue, which is associated with decreased arterial lipid deposition in mice [79].

The Wnt pathway can be therapeutically targeted at multiple points, and diverse strategies can be utilized. However, the major challenge results from the ubiquity of the Wnt pathway. This complex pathway plays a pivotal role in development and many other physiological processes; therefore, altering the normal Wnt pathway may result in detrimental side effects. Tissue-specific inhibition of the Wnt pathway could present a promising approach; however, in general, this approach currently remains at an early stage, and complexity exists in identifying a clinically feasible target. Similar challenges arise when considering strategies that might be applied to modulate the global Wnt pathway. Although small molecule inhibitors represent a potential therapeutic approach, they lack sufficient efficacy and specificity for use in humans [64,80,81]. Wnt ligands can form heterodimers that exert unique biological responses [82], and therefore any approach that proposes the use of recombinant Wnt proteins must consider potential impacts, including any unexpected impacts on malignancy [50].

## 8. Conclusions

Wnt signaling regulates the pathophysiology of many human diseases, including atherosclerosis. This unique pathway has enormous impacts on endothelial dysfunction, which initiates the pathological progression of atherosclerosis. In the later phases of atherosclerosis development, the Wnt pathway facilitates the migration of VSMCs to the intimal region and triggers macrophage mediated inflammation. Therapeutic targeting of the Wnt pathway may be promising; however, it may also interfere with normal Wnt-regulated physiological processes. Tissue-specific Wnt inhibition could be a better approach to avoid any off-target adverse effects. Endothelial-specific Wnt inhibition may provide protection against plaque formation. However, this approach needs further investigation to develop clinically feasible therapeutic compounds. Future research into the role of Wnt signaling in atherosclerosis has the potential to provide new scientific insights and development of real-world therapeutics.

## Data Availability

Not applicable.
